# Building Up Debondable Adhesion with Commercial Adhesives through Surface Modification of Substrate with Vitrimers

**DOI:** 10.1021/acspolymersau.6c00122

**Published:** 2026-08-21

**Authors:** Nien-Hsun Wu, Ying-Ling Liu

**Affiliations:** Department of Chemical Engineering, 34881 National Tsing Hua University, No. 101, Sec. 2, Kuang-Fu Road, Hsinchu 300044, Taiwan

**Keywords:** debondable adhesives, dynamic adhesion, vitrimers, surface modification, diketoenamine

## Abstract

ABSTRACT Debondable adhesion is highly attractive in the development of high-performance adhesives, particularly for sustainability initiatives requiring material recycling and reuse. Vitrimers, which possess dynamic features under specific conditions—typically at elevated temperatures—have been widely studied for the development of debondable adhesives. This work demonstrates an alternative approach to achieving debondable adhesion by establishing thin vitrimer layers on substrate surfaces via chemical modification. Poly­(ethylene terephthalate) (PET) films, surface-modified with diketoenamine (DKA)-based vitrimers, were prepared to demonstrate the proposed strategy. When a commercial epoxy AB glue was utilized as the adhesive, pristine PET films did not exhibit debondable behavior. In contrast, the vitrimer-modified PET films demonstrated debonding capabilities at 50 °C, evidenced by a reduction in lap shear strength from 8.2 ± 0.9 MPa to 3.3 ± 0.9 MPa. Furthermore, acid-catalyzed hydrolysis of the DKA layers in the adhered samples resulted in a reduction in lap shear strength from 4.9 ± 0.5 MPa to 1.8 ± 0.5 MPa. This study demonstrates a highly effective approach for engineering debondable interfaces that is readily applicable to commercial adhesive systems.

## Graphical abstract

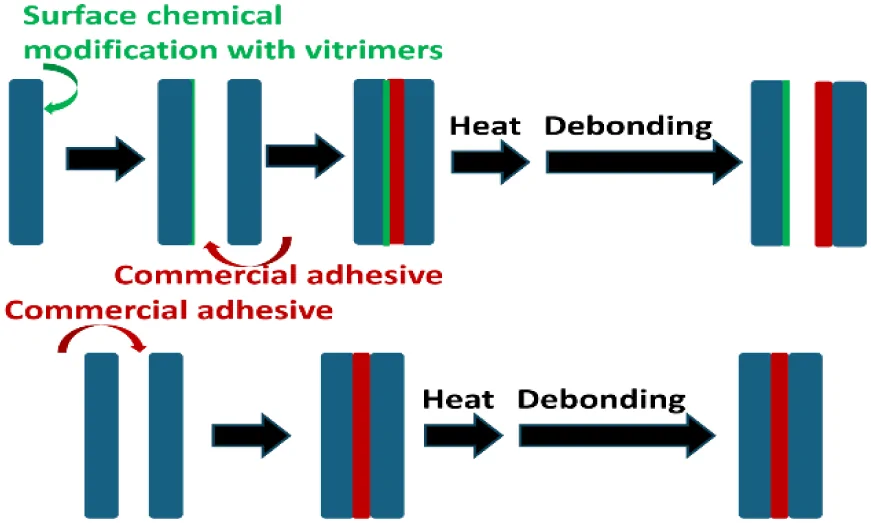

## Introduction

Vitrimers have emerged as a highly promising class of cross-linked polymers characterized by associative covalent adaptable networks (CANs).^1–3^ Owing to the dynamic nature of the chemical bonds within CANs, vitrimers exhibit reprocessing and recycling capabilities that are unattainable in conventional cross-linked thermosetting resins. Beyond structural design, synthetic methodology, and fundamental property investigations,^4–7^ the application of vitrimers as sustainable alternatives to conventional thermosets has garnered significant attention. For instance, vitrimer-based 3D-printed architectures have demonstrated remarkable repairability.^8^ Furthermore, vitrimer-based polymer electrolytes have been employed in lithium batteries to impart self-healing properties and enhance long-term charge–discharge cycling stability.^9–12^ Additionally, membranes fabricated from block copolymer vitrimers leverage dynamic bond exchange for nanoscale morphological control, resulting in improved flux and separation efficiencies.^13^

Beyond their use as matrix materials, thermosetting resins are widely utilized in adhesive applications.^14,15^ In most cases, high-performance adhesives provide robust bond strength between two adhered surfaces. However, when dismantling is required, debonding these materials can be exceedingly difficult. As sustainability issues become increasingly significant, debondable adhesives have attracted immense interest to facilitate convenient material recycling and reuse.^16–18^ Vitrimers exhibit significant stress relaxation at temperatures above their glass transition temperature (*T*g) and topology-freezing transition temperature (*T*v) due to the activation of bond-exchange reactions.^1,19^ Consequently, vitrimers have been extensively investigated for the development of debondable adhesives.^20^ Pioneering studies leveraged the reprocessing features of vitrimers to demonstrate repairable and reusable adhesives.^21–25^ Zhang et al.^21^ reported that Fe^3+^-cross-linked, carboxylic acid-containing supramolecular polymers demonstrate thermally induced declines in shear strength and debonding capabilities. Nevertheless, the relatively low temperatures of about 30–40 °C required for strength reduction and debonding may limit their practical applications. Du Prez and coworkers^26^ reported debondable epoxy adhesives utilizing reversible hardeners. The bond strength decreased significantly at 200 °C and retained approximately 35% of its original strength at 120 °C. Hence, the extent of the bond strength decline was highly dependent on both temperature and exposure time. Furthermore, modifying the dynamic bonds within the CANs of vitrimers can tune the temperatures required for bond-exchange reactions and network dynamics. Consequently, vitrimer-based debondable adhesives possessing a wide range of working and debonding temperatures have been reported.^27–33^ In addition to exchangeable covalent bonds, polyrotaxane-based movable networks have also been utilized in the development of dismantlable and recyclable adhesives, as reported by Ito and coworkers in 2026.^34^ Because the surface properties of substrates strongly dictate adhesion performance, De la Flor et al.^35^ recently reported improvements in the reversible adhesion of vitrimer adhesives through surface engineering. Similar to conventional adhesives, the surface properties of the substrates and the interfacial interactions between the adhesive and the substrate remain critical for the performance of vitrimer-based systems.

The literature discussed above has primarily focused on the use of vitrimers as alternatives to conventional adhesives. Most research efforts have concentrated on debonding mechanisms and the conditions required to trigger them. Nevertheless, other critical properties essential for practical adhesives, such as intrinsic adhesion strength, mechanical robustness, and wetting behavior on various substrates, are often overlooked. Consequently, significant challenges remain in translating these vitrimer-based debondable adhesives into practical applications. Alternatively, an approach that achieves debondable adhesion while utilizing commercially available adhesives would be highly practical and immediately applicable. In this work, we report a novel concept: introducing debonding and reworking capabilities at the substrate interface rather than within the adhesive matrix itself. Vitrimer structures were incorporated onto substrate surfaces via chemical modification to render the interfaces dynamic and adaptable. These incorporated vitrimer layers possess nanometer-scale thicknesses, thereby preserving the bulk properties of the adhered materials. Consequently, conventional adhesives can be applied to these surface-modified materials using standard, existing procedures. The proposed approach is demonstrated by chemically constructing diketoenamine (DKA)-based CANs^36,37^ on poly­(ethylene terephthalate) (PET) films through a multistep reaction sequence, thereby achieving both thermally triggered and catalytic hydrolysis-induced debonding capabilities. When a commercial adhesive is applied, the dynamic nature of the chemically modified vitrimer layer imparts debondable properties to the bonded interface. Using a conventional epoxy AB glue for adhesion tests, the debonding features of the vitrimer-modified surfaces were successfully triggered by either thermal treatment at 50 °C or acid-catalyzed hydrolysis, standing in stark contrast to the permanent adhesion observed on unmodified substrates. Overall, this work presents a highly effective strategy for leveraging vitrimer chemistry to engineer debondable adhesive systems that are directly compatible with existing commercial products.

## Experimental Section

### Materials

PET films were received from Dayu Plastic Corporation (Taiwan). Dimedone (>99%, TCI), *N*,*N*′-dicyclohexylcarbodiimide (DCC, 99%, Thermo Fisher), 4-dimethylaminopyridine (DMAP, 99%, Thermo Fisher), methacrylic acid (MAA, 99%, Thermo Fisher) were used as received. Tris­(2-aminoethyl)­amine (TREN, >98%, Acros) was stored with molecular sieves (4 Å) for drying prior to use. The used epoxy AB glue was received from Nan Ya Plastics Company (Taiwan).

### Instrumental Methods

Fourier transform infrared (FTIR) spectra were recorded on a PerkinElmer Spectrum Two spectrometer equipped with an attenuated total reflectance (ATR) accessory, using 32 scans at a resolution of 4 cm^–1^. X-ray photoelectron spectroscopy (XPS) measurements were conducted using a ULVAC-PHI 5000 VersaProbe II system at a radiation wavelength of 887 nm. For the thermally induced debonding tests (PET-DKA/wood samples), adhesion strengths were measured using an Instron 34TM-50 universal testing machine equipped with a 3119–615 environmental testing chamber. For the acid-catalyzed debonding tests (PET/PET-DKA samples), mechanical tests were performed on an Instron 5543 universal testing machine. Measurements of the adhered aluminum sheets were also conducted using the Instron 5543 system. Prior to bonding, the aluminum sheets were sequentially polished with P180 and P400 sandpaper. All mechanical measurements were performed at a crosshead speed of 5 mm min^–1^. Water contact angle measurements were conducted with a machine from First Ten Angstroms (Model: FTA1000B Class). An ozone generator (Model KI-10 from Three Oxygen Company (Taiwan)) was utilized for generation of O_3_/O_2_ mixture stream. The O_3_ generation capacity is 10 g h^–1^ at an O_2_ flow of 2.33 L min^–1^.

### Preparation of Diketoenamine-Based Vitrimer-Modified PET Films (PET-DKA)

PET films were washed with toluene and acetone, and then dried at 60 °C under vacuum. The cleaned PET films were immersed in toluene and the reactor was cooled with an ice bath. After being purged with an argon gas stream (6 L min^–1^ for 5 min), a stream of O_3_/O_2_ mixture (6 L min^–1^) from the ozone generator was applied to the reactor under bubbling for 10 min. The reactor was then purged again with an argon gas stream (6 L min^–1^ for 5 min). The PET films were draw out and dried at 60 °C under vacuum to give the sample of ozone-PET.

Ozone-PET were charged into a solution of MAA in toluene (20 mL MAA in 300 mL toluene). The reaction system was purged with an argon gas stream (6 L min^–1^ for 30 min), and then reacted at 110 °C for 3 h under refluxing. The samples were draw out, washed with toluene under ultrasonication for 3 times, washed with tetrahydrofuran (THF) under the same manner, and then draw out and dried at 60 °C under vacuum to give the sample of PET–PMAA.

PET–PMAA films were charged into a solution of dimedone (7.35 g), DMAP (9.16 g), and DCC (12.38 g) in *N*,*N*-dimethylforamide (DMF, 50 mL). After being reacted at room temperature for 6 h, the samples were draw out, washed with ultrasonication in acetone, THF, 1.0 N HCl_(aq)_, and pure water sequentially. The samples were then dried at 60 °C under vacuum to give PET-TK.

PET-TK was put in a solution of TREN in ethanol (5% v/v). After being reacted at 60 °C for 6 h, the samples were draw out and washed with ethanol under ultrasonication. The obtained samples were stand at room temperature for 12 h and then dried at 60 °C under vacuum for 12 h to give PET-DKA.

### Debonding Behavior Observation and Lap Shear Tests

PET films (pristine PET and PET-DKA) and wood sheets were cleaned with methanol and dried at 50 °C for 30 min. A PET film and a wood sheet were bonded with the epoxy AB glue with an adhered area of 10 × 10 mm^2^. After being loaded with an 11.8 N gravity force, stated at room temperature for 5 min, and then heated with a heat gun. The debonding observation was conducted once every 10 °C increase in temperature. The tests on the adhered PET-DKA/wood samples were conducted with the same manner. The samples for lap shear measurements at different temperatures were prepared with the same manner mentioned above. The adhered areas were adjusted to be 2.5 × 10 mm^2^. Mechanical tests on the samples were carried out at 25 and 50 °C, respectively. Experimental results were recorded with 3 different samples to give averaged values and measurement errors.

### Acid-Catalyzed Debonding Tests

The adhered PET/PET and PET/PET-DKA samples with adhered areas of 2.5 × 10 mm^2^ were prepared with the same manner mentioned above. The samples were immersed in a 1.0 N HCl_(aq)_ solution at 50 °C for 2 h. The samples were draw out, washed with pure water, and then dried at room temperature under vacuum. Mechanical tests on each type samples were conducted at 25 °C for 3 times to obtain averaged values and measurement errors.

## Results and Discussion

Surface functionalization of PET films was achieved through a multistep reaction sequence to incorporate diketoenamine (DKA)-based CANs onto the substrates (Figure 1). First, ozone treatment was utilized to introduce peroxide and hydroperoxide groups onto the PET surfaces. These species subsequently served as initiators for the surface-initiated radical polymerization of methacrylic acid (MAA).^38^ Next, dimedone was reacted with the carboxylic acid groups of the grafted poly­(methacrylic acid) (PMAA) chains to yield triketone groups. Finally, these triketone intermediates were cross-linked with the primary amine groups of tris­(2-aminoethyl)­amine (TREN)^36,37^ to generate the DKA-based associative CANs on the PET surfaces.

**Figure 1:**
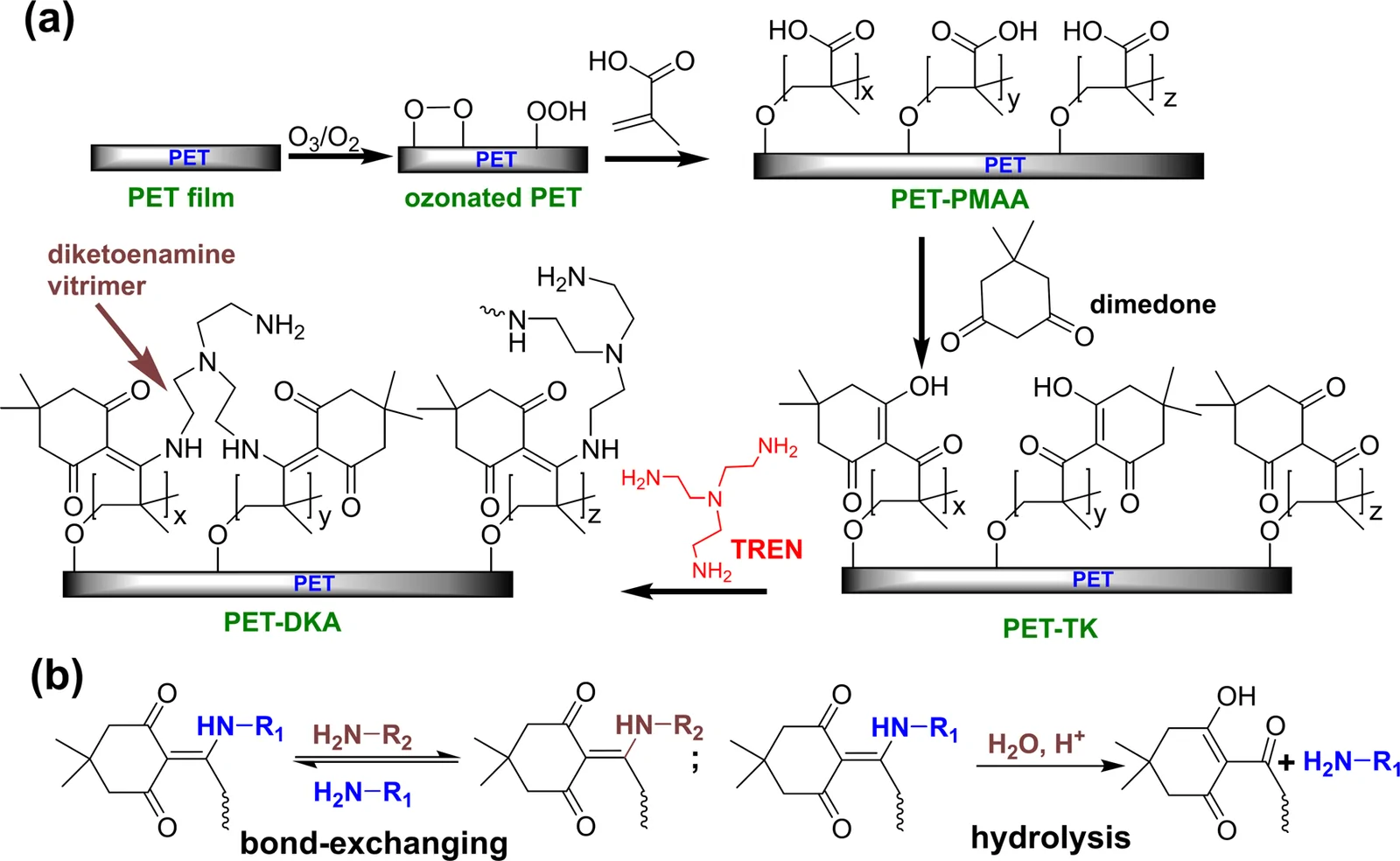
(a) Chemical reaction routes for building up diketoenamine-based vitrimers on PET film surfaces; (b) Bond exchanging and hydrolysis reactions of diketoenamine groups.

Changes in the chemical structure of the PET film surfaces following each reaction step were monitored using ATR-FTIR spectroscopy (Figure 2). Ozone treatment did not result in discernible changes in the ATR-FTIR spectra because the oxidation occurred only in the outermost surface layer of the PET films. Following the grafting of PMAA chains, new absorption peaks appeared at 1690 and 2588 cm^–1^ in the spectrum of PET–PMAA, corresponding to the stretching vibrations of the −COOH groups.^39^ Subsequent conversion of the −COOH groups to triketone groups led to a decrease in the −COOH absorption intensities and the emergence of characteristic triketone absorptions at 1757, 1648, 1560, and 1042 cm^–1^ for PET-TK.^37^ These triketone absorptions disappeared following the cross-linking reaction with TREN. The resulting PET-DKA sample exhibited only the characteristic absorptions of the diketoenamine (DKA) groups at 1639 and 1546 cm^–1^, assigned to the C=O and C=C stretching vibrations, respectively. Further chemical characterization of the surface-modified PET films was performed using XPS. First, surface oxidation via ozone treatment increased the total oxygen content from 21.7 at % (pristine PET) to 24.5 at % (O3-PET), and the C–O linkage fraction increased from 28.4% to 32.7%. In the C_1s_ core-level XPS spectrum of PET–PMAA (Figure 3a), the presence of the grafted PMAA chains was confirmed by a new peak at 285.8 eV, assigned to the −COOH carbon. The triketone groups in PET-TK were identified by a peak at 287.4 eV in the C_1s_ spectrum, along with peaks at 531.4 and 532.2 eV in the O 1s spectrum (Figure 3b). Finally, the successful formation of the DKA groups was verified by the appearance of N 1s signals at 399.6 (C–N) and 400.7 eV (C=N) in the N_1s_ core-level spectrum of PET-DKA (Figure 3c).^40^

**Figure 2:**
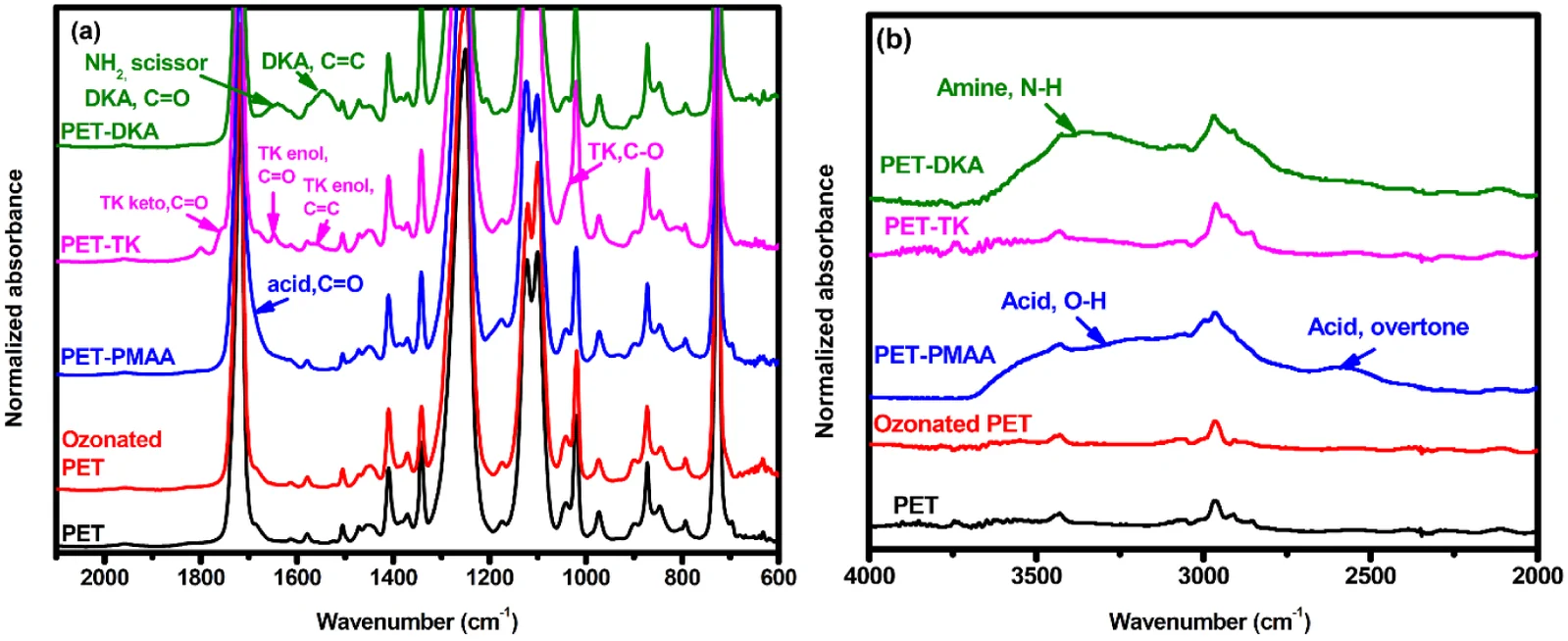
ATR-FTIR spectra recorded on the surface-modified PET samples: (a) the spectra in 600–2100 cm^–1^ and (b) the spectra in 2000–4000 cm^–1^.

**Figure 3:**
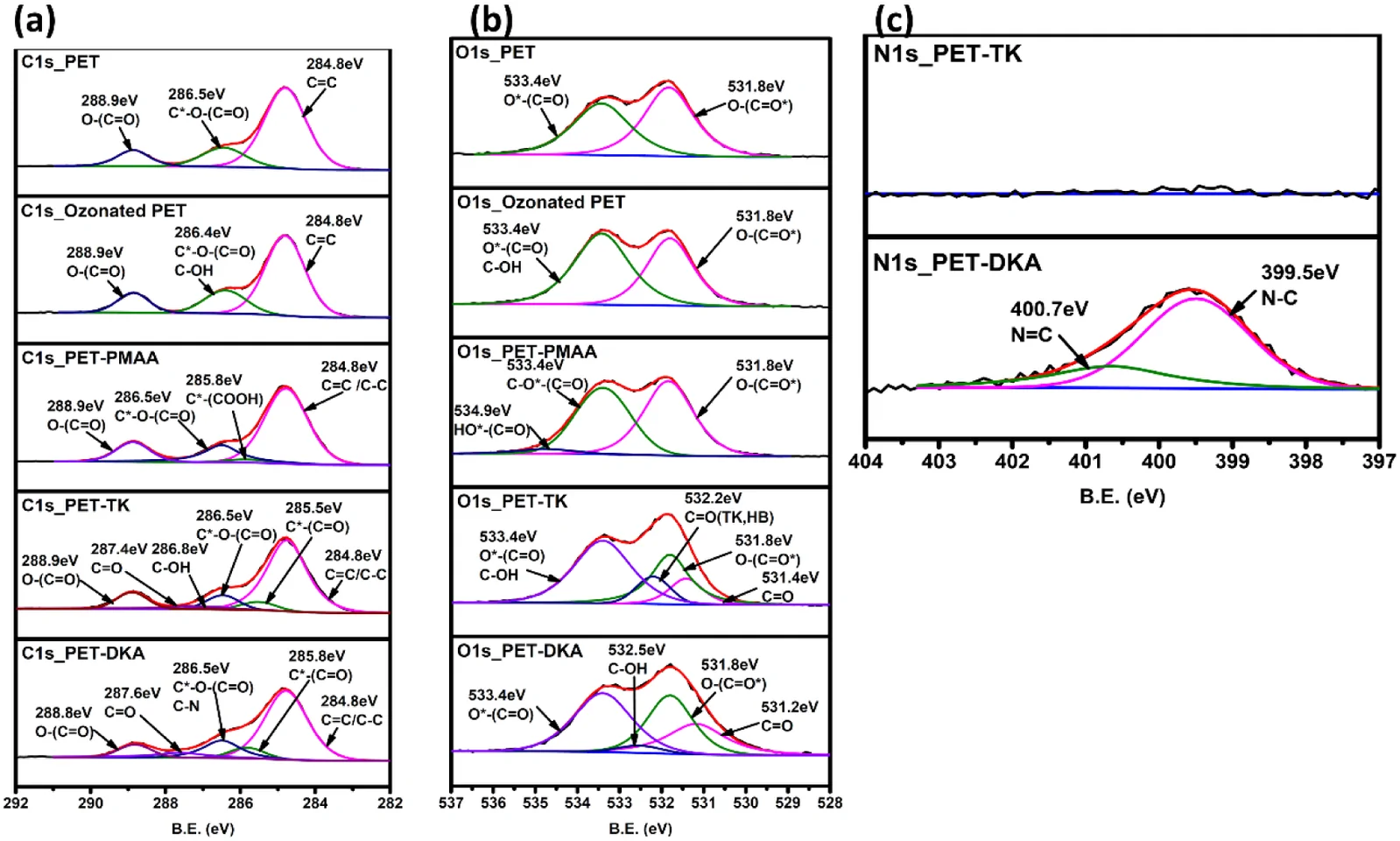
XPS spectra recorded on the surface-modified PET samples: (a) C_1s_ core-level spectra, (b) O_1s_ core-level spectra and (c) N_1s_ core-level spectra.

It is noteworthy that the PET-DKA sample still exhibited distinct PET signals in the XPS spectra, suggesting that the thickness of the incorporated DKA layer is less than a few tens of nanometers (the typical sampling depth of XPS). Furthermore, changes in the chemical structure associated with the surface modifications were evaluated via water contact angle (WCA) measurements. The pristine PET film exhibited a WCA of approximately 75°. Following the sequential modifications, the WCAs measured for PET–PMAA, PET-TK, and PET-DKA were 60°, 83°, and 69°, respectively. These variations are highly consistent with the chemical nature of the newly introduced functional groups. Notably, the pristine PET and the final PET-DKA films did not exhibit a significant difference in their overall surface wettability.

The surface-modified PET featuring diketoenamine (DKA)-based CANs (PET-DKA) was subsequently subjected to debondable adhesion tests. Unlike previous studies that utilized specially designed and synthesized vitrimer-based adhesives,^21–35^ this work employed a commercial two-part epoxy to demonstrate that the debonding capabilities originate solely from the CAN-based layer chemically grafted onto the substrate surface. Both the pristine PET and PET-DKA substrates were bonded to wood pieces using the commercial adhesive. The bonded samples were then subjected to a static load of approximately 11.8 N during heating. The pristine PET/wood sample did not debond even at 80 °C, demonstrating the robust thermal stability of the employed epoxy adhesive. In contrast, the PET-DKA/wood sample successfully debonded at approximately 55 °C (Figure 4). This observed debonding temperature falls well within the dynamic temperature range previously reported for DKA-based vitrimers.^36,37^ To determine the adhesion failure mode, both the PET-DKA substrate and the wood piece recovered from the debonding test were subjected to spectral characterization. In the ATR-FTIR measurements (Figure 5), the wood surface exhibited a spectrum highly similar to that of the cured epoxy adhesive. Moreover, the spectra recorded for the original PET-DKA and the PET-DKA sample postdebonding were nearly identical. These results indicate that adhesion failure occurred strictly at the interface between the PET-DKA layer and the epoxy adhesive. Almost all of the epoxy adhesive remained bonded to the wood surface, leaving virtually no residue on the PET-DKA surface. Therefore, the debonding behavior is exclusively attributed to the dynamic network of the DKA-based vitrimers on the PET-DKA surface, rather than to cohesive failure within the epoxy adhesive itself. This conclusion was further corroborated by XPS analysis. The N_1s_ core-level spectra of the original PET-DKA and the postdebonding PET-DKA sample were identical. While the pristine epoxy adhesive exhibited two distinct N_1s_ signals assigned to aniline (400.1 eV) and C–N (399.2 eV) environments, these signals were entirely absent from the PET-DKA surface after the debonding test. Conversely, the wood surface after the debonding test continued to exhibit signals corresponding to the epoxy adhesive, confirming that it remained covered by the polymer matrix. Moreover, a small signal of diketoenamine at 400.7 eV was also observed, to suggest some diketoenamine might be peeled off from the PET-DKA surface due to the strong adhesion power of the epoxy glue. Nevertheless, as the sample depth of XPS analysis is in nanometers, the amount of peeled off diketoenamine could be very small. The experimental results indicate the diketoenamine-based layer serving as an effectively dynamic and nonsticking layer for the glue under debonding operation. For quantitative evaluation, the bonded PET/wood and PET-DKA/wood samples, with an overlap area of 2.5 × 10 mm^2^, were subjected to tensile lap shear tests. During measurements at 25 °C, both sample types fractured within the PET substrates rather than at the bonded interface. This behavior is attributed to the robust intrinsic adhesion strength of the applied epoxy, as evidenced by bonded aluminum control samples which exhibited a lap shear strength of 16.1 ± 0.6 MPa. Consequently, the recorded failure strengths (7.0 ± 0.8 and 8.2 ± 0.9 MPa for PET/wood and PET-DKA/wood, respectively) reflect the mechanical tensile limit of the PET films rather than the true adhesion strength of the epoxy joint. In contrast, the PET-DKA/wood samples tested at 50 °C failed directly at the bonded interface, yielding a significantly reduced lap shear strength of 3.3 ± 0.9 MPa. This pronounced decrease in adhesion strength for the epoxy applied on the vitrimer-modified surface is directly attributed to the thermally activated dynamic behavior of the diketoenamine (DKA)-based networks. These results demonstrate that surface modification with vitrimers is a highly effective approach for engineering debondable interfaces that are fully compatible with conventional adhesives.

**Figure 4:**
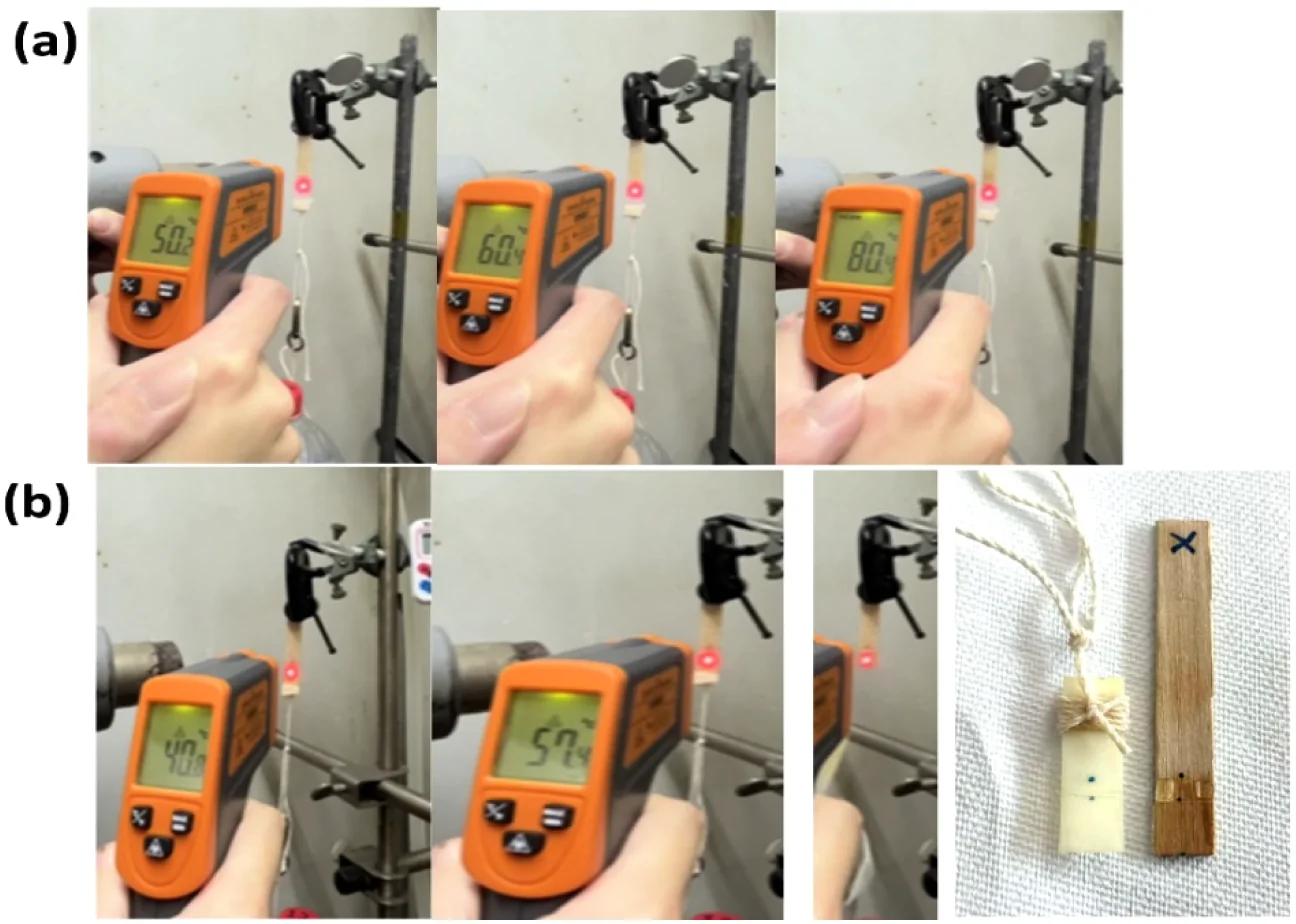
Pictures taken in the thermally induced debonding tests. (a) The PET-DKA/wood sample not debonding until 80 °C and (b) the PET-DKA/wood sample debonding at about 55 °C.

**Figure 5:**
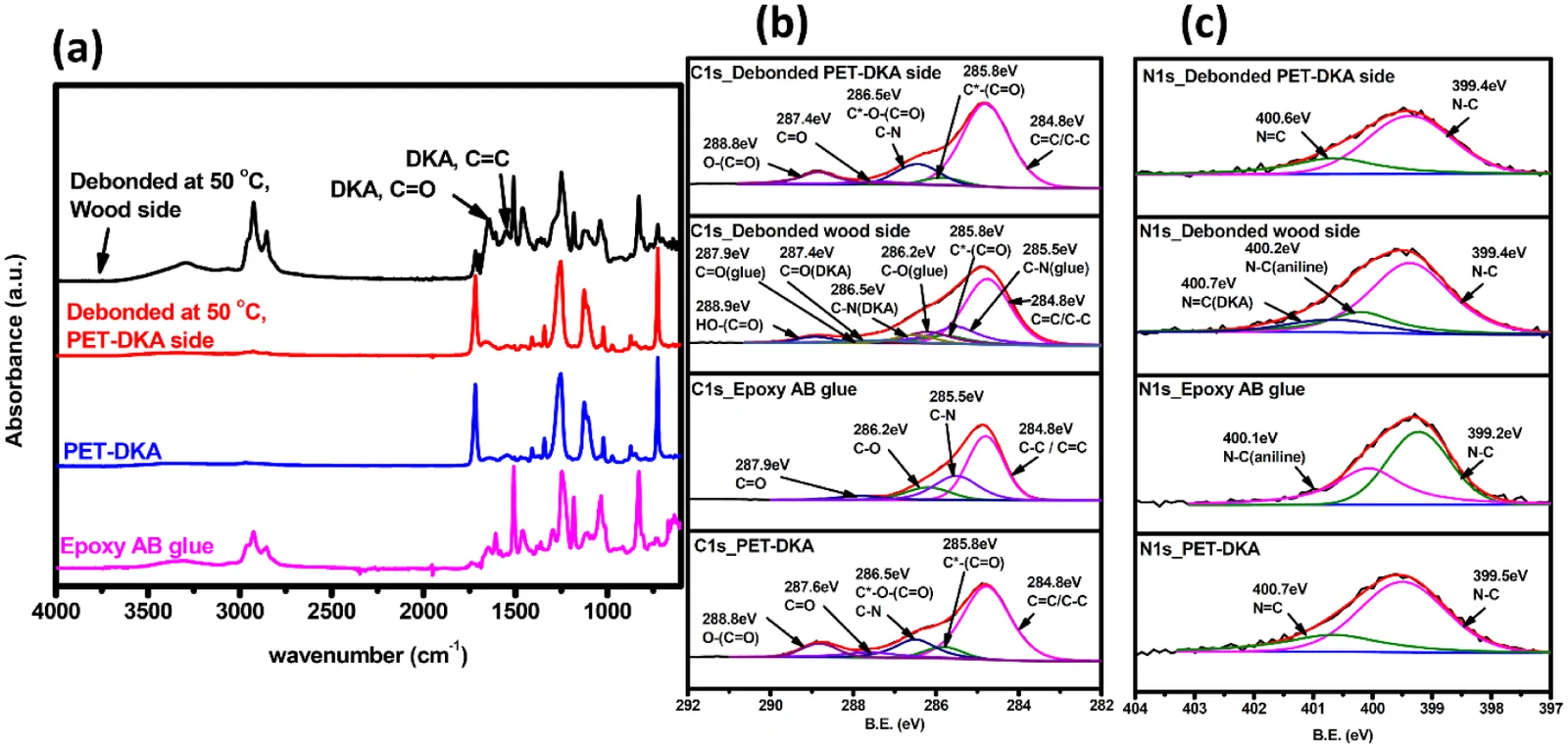
(a) ATR-FTIR spectra, (b) XPS C_1s_ core-level spectra and (c) XPS N_1s_ core-level spectra recorded on the surfaces of PET-DKA/wood debonded samples.

In addition to their temperature-responsive behavior, cross-linked diketoenamine (DKA) vitrimers also undergo acid-catalyzed hydrolysis.^36,37^ This feature was exploited to debond the bonded PET/PET-DKA joints (overlap area: 2.5 × 10 mm^2^). The reference sample (bonded PET/PET) exhibited lap shear strengths (measured at 25 °C) of 5.0 ± 0.7 and 5.0 ± 1.1 MPa before and after immersion in 1.0 N HCl_(aq)_ for 1 h at 50 °C, respectively. In contrast, the lap shear strength of the bonded PET/PET-DKA sample decreased significantly from 4.9 ± 0.5 to 1.8 ± 0.5 MPa following the acid treatment (Figure 6b). The acidic conditions catalyzed the hydrolysis of the DKA vitrimer networks, consequently resulting in the observed reduction in adhesion strength. As shown in the optical images (Figure 7a), both PET substrates recovered from the debonded PET/PET reference sample retained residues of the epoxy adhesive, indicating cohesive failure within the adhesive layer. Conversely, the PET/PET-DKA sample exhibited asymmetric interfacial fracture; the adhesive remained entirely on the unmodified PET side, leaving the PET-DKA side virtually residue-free (Figure 7b). Furthermore, the debonded surfaces were analyzed using ATR-FTIR spectroscopy (Figure 7c). Characteristic signals of the cured epoxy were observed only in the spectrum of the unmodified PET substrate and were completely absent from the PET-DKA spectrum. Therefore, the hydrolysis of the DKA layer on the PET-DKA substrate degrades its cross-linked structure, facilitating the detachment of the epoxy adhesive. The adhesion failure strictly occurred at the interface between the adhesive and the PET-DKA layer. These results confirm that the debonding behavior was triggered by the hydrolysis of the DKA-based vitrimer layer rather than the degradation of the epoxy adhesive itself. Ultimately, substrate surface modification is proven to be a highly effective strategy for engineering dynamic interfaces for debondable adhesion.

**Figure 6:**
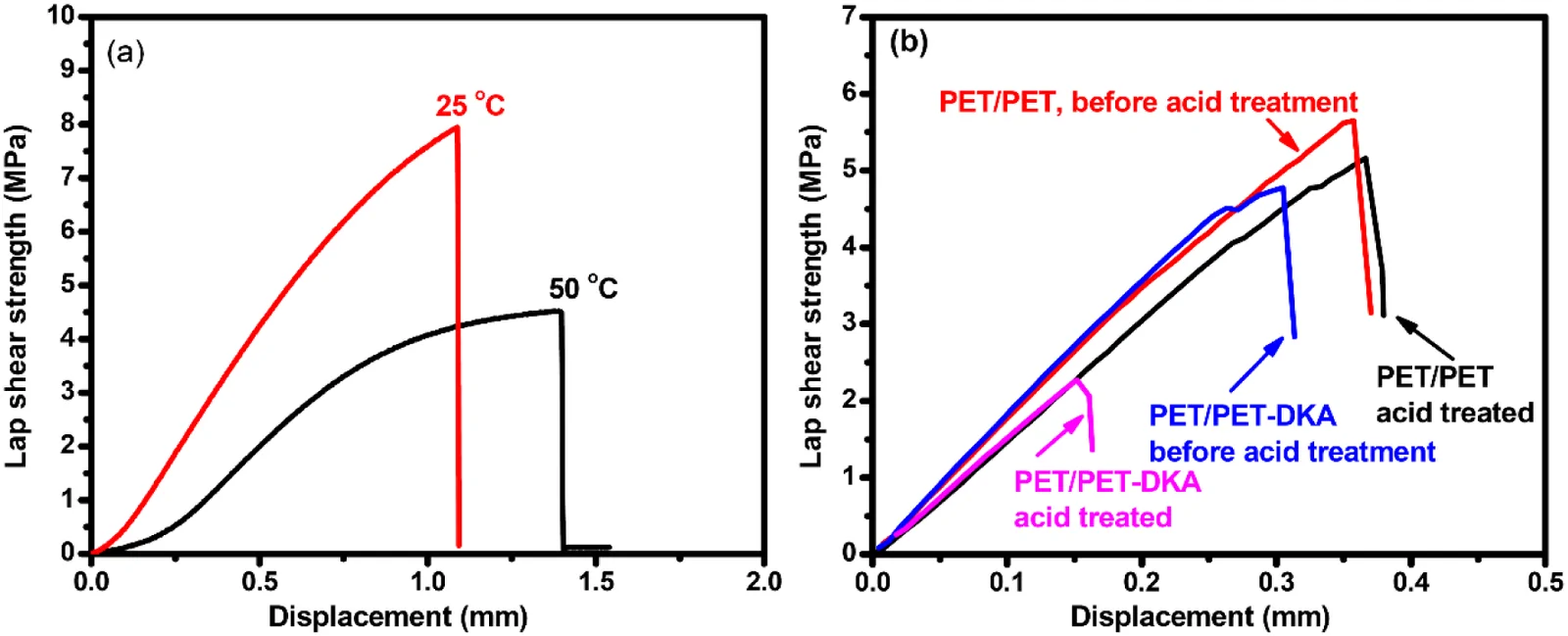
Lap shear strength tests on the adhered sample. (a) PET-DKA/wood samples at different temperatures and (b) PET/PET and PET/PET-DKA samples before and after acid treatments.

**Figure 7:**
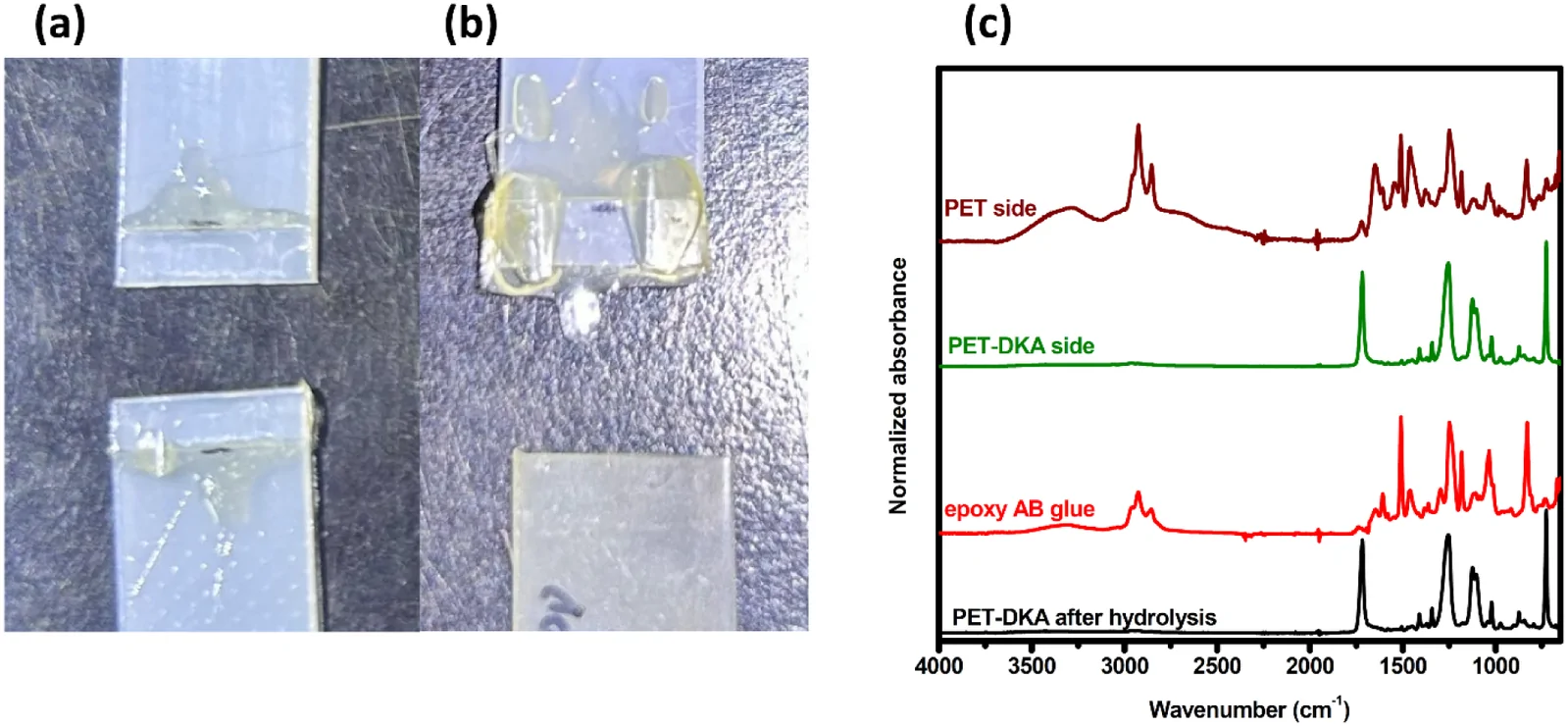
Photographs of hydrolyzed and debonded (a) PET/PET sample and (b) PET/PET-DKA sample; (b) ATR-FTIR spectra recorded on the surfaces of hydrolyzed and debonded PET/PET-DKA sample.

## Conclusions

In contrast to previous developments in vitrimer-based debondable adhesives, this work demonstrates an effective approach to engineering debondable interfaces by incorporating dynamic networks onto substrate surfaces via chemical modification. Using a diketoenamine (DKA)-based dynamic network as the modification layer and a commercial epoxy AB glue as the adhesive, this concept was successfully demonstrated on poly­(ethylene terephthalate) films. The resulting adhesion exhibited temperature-responsive behavior; the lap shear strength of the adhered samples decreased from 8.2 ± 0.9 to 3.3 ± 0.9 MPa when the temperature was increased from 25 to 50 °C. Furthermore, the adhesion strength could be reduced from 4.9 ± 0.5 to 1.8 ± 0.5 MPa following acid treatment, owing to the acid-catalyzed hydrolysis of the DKA-based networks. In future work, the conditions required for debonding can be tuned by varying the types and structures of the dynamic bonds within the vitrimers.
